# Immune-related adverse events with immune checkpoint inhibitors affecting the skeleton: a seminal case series

**DOI:** 10.1186/s40425-018-0417-8

**Published:** 2018-10-11

**Authors:** Kendall F. Moseley, Jarushka Naidoo, Clifton O. Bingham, Michael A. Carducci, Patrick M. Forde, Geoffrey T. Gibney, Evan J. Lipson, Ami A. Shah, William H. Sharfman, Laura C. Cappelli

**Affiliations:** 1 Department of Medicine, Division of Endocrinology, Diabetes & Metabolism, Johns Hopkins University School of Medicine, Baltimore, MD USA; 2 Department of Oncology, Sidney Kimmel Comprehensive Cancer Center, Johns Hopkins University School of Medicine, Baltimore, MD USA; 3 Department of Medicine, Division of Rheumatology, Allergy and Immunology, Johns Hopkins University School of Medicine, Baltimore, MD USA; 4 Department of Oncology, Georgetown Lombardi Comprehensive Cancer Center, Medstar Georgetown University Hospital, Washington D.C., USA

**Keywords:** Immunotherapy, Immune-related adverse events, Bone resorption, Fracture

## Abstract

**Background:**

The use of immune checkpoint inhibitors is increasing in cancer therapy today. It is critical that treatment teams become familiar with the organ systems potentially impacted by immune-related adverse events associated with these drugs. Here, we report adverse skeletal effects of immunotherapy, a phenomenon not previously described.

**Case presentations:**

In this retrospective case series, clinical, laboratory and imaging data were obtained in patients referred to endocrinology or rheumatology with new fractures (*n* = 3) or resorptive bone lesions (*n* = 3) that developed while on agents targeting PD-1, CTLA-4 or both. The average age of patients was 59.3 (SD 8.6), and five were male. Cancer types included melanoma, renal cell carcinoma and non-small cell lung cancer. All fracture patients had vertebral compression, and two of the three had multiple fracture sites involved. Sites of resorptive lesions included the shoulder, hand and clavicle. Biochemically, elevated or high-normal markers of bone resorption were seen in five of the six patients. Erythrocyte sedimentation rate was elevated in three of the four patients where checked.

**Conclusions:**

This case series represents the first description of potential skeletal adverse effects related to immune checkpoint inhibitors. These findings are important for providers caring for patients who experience musculoskeletal symptoms and may merit additional evaluation.

## Background

Immune checkpoint inhibitors (ICIs) are widely considered to be a therapeutic breakthrough for cancer. Antibodies targeting immunoregulatory molecules such as programmed death-1 (PD-1), its ligand PD-L1, and cytotoxic T-lymphocyte associated protein 4 (CTLA-4) are in widespread use for the treatment of lung, gastric, bladder, kidney, urothelial, head and neck, hepatocellular, and mismatch repair deficient/microsatellite instability-high cancers. These agents modulate host immune responses principally by activating cytotoxic T-cells that are responsible for tumor cell destruction [1]. As these therapies continue to demonstrate efficacy in clinical trials and, consequently, garner approval for an increasing number of indications, ICI use is expected to increase in the years to come.

Toxicities associated with ICIs – often referred to as immune-related adverse events (irAEs) – have been reported in nearly every organ system. The mechanisms that underlie irAE development are poorly understood, but are likely due to increased systemic inflammation caused by ICI therapy, resulting in autoimmune responses as well as dysregulation of T-cell self-tolerance [2]. More commonly recognized irAEs include colitis, hepatitis, pneumonitis, thyroiditis, hypophysitis and skin rash [3]. Rheumatologic irAEs have been reported including inflammatory arthritis, myositis, and polymyalgia rheumatica-like syndromes [4–8]. Absent from the literature to date are descriptions of ICI effects on the skeleton.

The important interaction between the immune system and bone is increasingly appreciated [9, 10]. Studies of pro-inflammatory states demonstrate that alterations in T-cell mediated cytokines favor bone resorption [11–16]. We therefore hypothesize that immune activation induced by ICIs may adversely impact T-cell-mediated skeletal remodeling, leading to bone erosion and/or diffuse loss. To our knowledge, this report represents the first case series describing skeletal irAEs associated with ICIs. Among six patients treated with ICIs, we observed two distinct skeletal phenotypes: 1) new-onset osteoporosis leading to fracture, and 2) localized bony resorption. Herein, we briefly describe each patient’s treatment history, irAE presentation, and clinical outcome.

## Case presentations

### Patients and methods

Included in this series are patients evaluated and treated at the Sidney Kimmel Comprehensive Cancer Center at Johns Hopkins Hospital who were referred to the endocrinology or rheumatology services for new skeletal issues (osteoporosis/osteopenia, pathologic fractures, and destructive or resorptive bone lesions) that arose during treatment with one or more ICIs, administered as standard-of-care or as a part of a clinical trial. Patient and tumor features including medical history, tumor histology, cancer therapies, and use of concomitant medications (including bisphosphonates or RANK ligand inhibitors) were collected. Risk factors for bone loss were gathered from clinical assessment and review of the electronic medical record including: focal bone radiation, family history of osteoporosis, tobacco or alcohol abuse, renal disease and prolonged corticosteroid use. Laboratory data obtained as part of clinical care included markers of bone resorption and formation, inflammatory markers, serum calcium and phosphorus, parathyroid hormone, and 25-hydroxy-vitamin D. Radiologic imaging data were obtained as clinically indicated. Where available, pathologic data from bone biopsies were reviewed. Patients with preexisting pathologic fracture(s), metabolic bone disease, osteoporosis, inflammatory arthritis or other autoimmune diseases were excluded.

### Results

Six patients with skeletal irAEs were identified - three with new osteoporotic fractures and three with focal bone resorptive lesions. Patient features are summarized in Table 1. Patients were 51–75-years old at the time of development of the skeletal event, and five were male. Cancer diagnoses included: metastatic melanoma (*n* = 4), renal cell carcinoma (*n* = 1), and non-small cell lung cancer (*n* = 1). Four patients were treated with anti-PD-1 monotherapy, while two patients received ipilimumab (anti-CTLA-4) plus nivolumab combination ICIs. Only one of the six patients experienced additional irAE not related to the musculoskeletal system. All patients with fractures (*n* = 3) experienced the skeletal event within the vertebral column; one had additional rib and pelvic fractures. No patient with fractures had osteoporosis by dual -energy X-ray absorptiometry (DXA) definition (i.e., all T-scores > − 2.5). All three patients with destructive or resorptive bone lesions had concomitant inflammatory arthritis in separate joints that developed while receiving ICI therapy. No patient in this series developed both skeletal phenomena.

**Table 1:** Patient demographics

Patient	Skeletal AE	Age	Sex	Race	BMI	Tumor type and stage	Treatment Regimen	Bone Metastases?	Other irAEs
1	Compression fractures of T6, T7, T10, T11, and T12; rib and pelvic fractures	75	M	C	19	Melanoma Stage IV	Pembrolizumab	No	None
2	Compression fractures, T6–12, L1	52	M	C	27	Melanoma Stage IV	Nivolumab	Yes	None
3	Compression fracture, T11; lumbar osteomalacia	58	M	C	29	Melanoma Stage IV	Pembrolizumab	No	None
4	Resorptive bone lesion, left shoulder	60	M	C	24	Melanoma Stage IV	Ipilimumab/nivolumab	No	Pneumonitis, hypophysitis, inflammatory arthritis
5	Resorptive bone lesion, right wrist	60	F	C	26	Renal cell carcinoma Stage IV	Nivolumab	Yes	Inflammatory arthritis
6	Resorptive bone lesion, right clavicle	51	M	C	25	Non small cell lung cancer Stage II	Ipilimumab/nivolumab	Yes	Inflammatory arthritis

(*AE*) Adverse event, (*irAEs*) Immune-related adverse events

#### Spontaneous fractures / new onset osteoporosis

Three patients without a prior diagnosis of osteoporosis were identified with new vertebral compression fractures and deformities that occurred or evolved during the course of immunotherapy (patients 1, 2, 3). No risk factors for bone loss or osteoporosis preceding ICI treatment were identified in any patient. No patient with fracture or newly-identified bone loss sustained additional irAEs.

### Patient 1

**Fig. 1:**
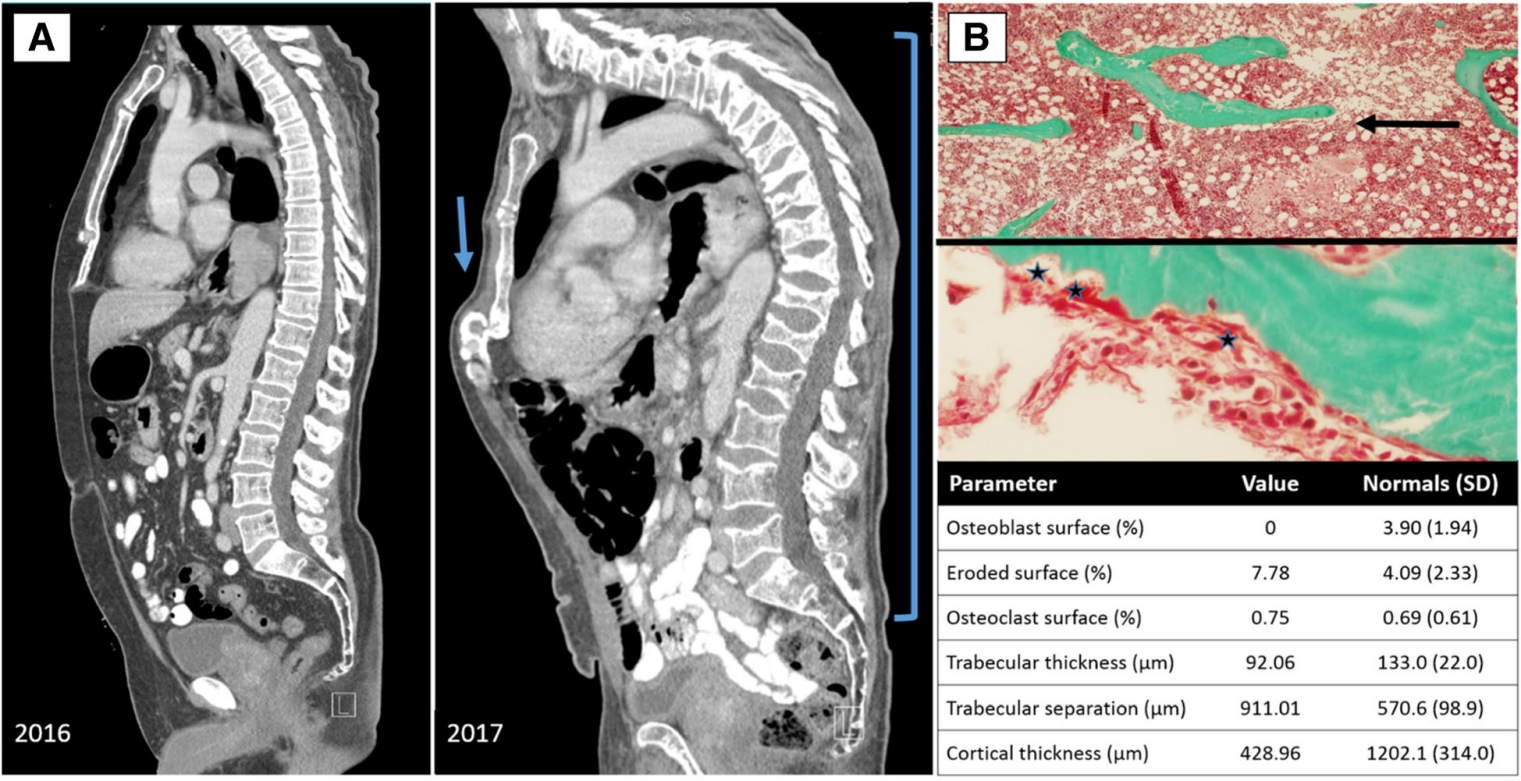
**a** Patient 1, chest CT scan demonstrating new thoracic and lumbar compression fractures, evolving over one year on immunotherapy, 2016 compared to 2017 (bracket); new sternal deformity in the setting of marked kyphosis with compensatory sternal compression (arrow). **b** Patient 1, transiliac bone biopsy photographed at 400×; top panel illustrates thin trabeculae and limited connectivity, consistent with osteoporosis (arrow); middle panel demonstrating increased osteoclastic activity at the sites of three Howship’s lacunae, or resorption pits (stars); bottom panel outlines histomorphometry parameters including increased eroded surface as well as decreased trabecular and cortical thickness but increased trabecular separation consistent with low bone mass

Patient 1 is a 75-year old male, who was originally diagnosed with stage IIIB, BRAF-negative melanoma of the upper back and left axillary lymph node (LN) involvement in 2012, treated with wide local excision (Breslow thickness: 2.9 mm) and axillary LN dissection. The patient received adjuvant therapy with a GM-CSF secreting allogeneic melanoma cell vaccine for 3-years, but developed recurrent disease at the right buttock, inguinal nodes and lung in 2015, and was treated with first-line pembrolizumab monotherapy. He received 25 total doses and sustained a radiologic complete response to therapy by RECIST 1.1 v.5.0. After 20 doses of pembrolizumab therapy, he developed acute back pain; a contrast-enhanced MRI of the full spine demonstrated multiple, non-traumatic vertebral compression fractures, rib fractures, and as well as pelvic fractures sustained during therapy, without bone metastases. ICI therapy was continued, however he developed additional compression fractures and more profound vertebral wedging (Fig. 1a), prompting discontinuation of pembrolizumab after 18-months of therapy. The patient’s biochemical workup was unremarkable. His degree of active bone resorption, as measured by C-telopeptide levels (CTX, Table 2) were elevated despite three-weeks of alendronate use prior to appointment. Bone density at the hip (lumbar spine excluded in the setting of fracture) demonstrated osteopenia only. Histomorphometry from transiliac bone biopsy (Fig. 1b) revealed bone resorption (increased eroded surface, osteoclast surface) and bone loss (reduced trabecular and cortical parameters). Given the patient’s continued bone loss on oral bisphosphonate, he received one infusion of intravenous bisphosphonate (zoledronic acid), underwent multiple kyphoplasty procedures, and permanently discontinued pembrolizumab. At present, his melanoma continues to be in complete remission 35-months after commencement of pembrolizumab, and after therapy has been held for 20-months. The patient continues to receive IV bisphosphonate yearly in the form of zoledronic acid.

**Table 2:** Skeletal characteristics and clinical course

Patient	Treatment and clinical course	Dual energy X-ray absorptiometry (DXA) T-scores^a^	Additional imaging	Bone resorption and formation markers/biochemical indices	Inflammatory markers	Biopsy data
1	Intravenous zoledronic acid infusions NED; progressive back pain requiring kyphoplasty	T-scores: Lumbar spine, −2.1 Left femoral neck, − 1.2 Left total hip, −0.4 Right femoral neck, − 1.7 Right total hip, − 0.8	MRI: Multilevel compression fractures of T12-L5 vertebral bodies; bilateral posterolateral rib fractures; multiple nondisplaced pelvic fractures superimposed on underlying osteopenia.	CTX: 1038 pg/mL (↑) bsALP: 11.6 μg/L Ca: 9.5 mg/dL 25OHD: 48 ng/mL	CRP: 1.0 mg/dL (↑) ESR 12 mm/h	Transiliac bone biopsy: Reduced cortical and trabecular thickness; no osteoblastic activity; increased eroded surface and osteoclastic resorptive activity.
2	Denosumab q6mo Progressive metastatic disease requiring hospice care; now deceased	T-scores: Left femoral neck, − 1.5 Left total hip, 0.1	VFA: Multiple wedge deformities 16.8% in T6, 13.6% in T7, 11.2% in T8, 18% in T9, 4.1% in T10, 6.9% in T12, 13.2% in L1. CT chest 1/17: New compression deformity of the superior endplates of T2-T5 vertebral bodies	CTX: 537 pg/mL bsALP: 13.1 μg/L Ca: 8.9. mg/dL 25OHD: 37 ng/mL	N/A	N/A
3	Conservative management with calcium and vitamin D optimization NED, pain free	T-scores: L-spine, − 0.9 Left femoral neck, − 1.8 Left total hip, − 1.2	CT scan: T12 compression fracture with evolving compression deformity at T11; biconcave deformities of lumbar vertebrae	CTX: 335 pg/mL bsALP: 8.7 μg/L Ca: 9.4. mg/dL 25OHD: 18 ng/mL (↓)	N/A	N/A
4	Systemic oral steroids Adalimumab NED; improved inflammatory arthritis but progressive left shoulder pain and lack of mobility; no additional resorptive lesions	T-scores: L-spine, − 1.0 Left femoral neck, − 1.2 Left total hip, 0.2 1/3 radius, − 1.9	MRI shoulder: Severe erosive changes of the glenohumeral articulation	CTX: 589 pg/mL (↑) bsALP: 18 μg/L (↑) Ca: 9.4. mg/dL 25OHD: 52 ng/mL	CRP: 2.7 mg/dL (↑) ESR: 69 mm/h (↑)	No evidence of melanoma (S100, HMB-45 and melan-A stains negative). Trabecular bone with bone marrow fibrosis and a scattered, mixed inflammatory cell infiltration.
5	Systemic oral steroids Progressive, metastatic disease; limited response to steroids, currently in hospice	N/A	Hand X-ray: Loss of ossific densities related to the capitate and also hamate, loss of cortical outline of the capitate and hamate	CTX: 877 pg/mL (↑) bsALP: 24 μg/L (↑) Ca: 8.8 mg/dL 25OHD: 51.8	CRP: 13.5 mg/L (↑) ESR: 31 mm/h (↑)	N/A
6	NSAIDs, Intraarticular corticosteroids Progressive, metastatic disease; good rheumatologic response to steroids, currently on carboplatin pemetrexed and bevacizumab	T-scores: L- spine, 0.8 Left femoral neck, 0. 0 Left total hip, 0.8	MRI clavicle: Acromioclavicular joint arthrosis with disproportionate bone marrow edema affecting the distal, no visible fracture	CTX: 681 pg/mL (↑) bsALP: 17 μg/L (↑) Ca: 8.8 mg/dL 25OHD: 22 ng/mL (↓)	CRP: 2.0 mg/dL (↑) ESR: 32 mm/h (↑)	N/A

^a^T-score criteria by DXA: > − 1, normal bone density; − 1 to − 2.4, osteopenia; < − 2.5, osteoporosis

(*L-spine*) Lumbar spine, (*N/A*) Not applicable, (*NED*) No evidence of disease, (*CT*) computed tomography, (*MRI*) magnetic resonance imaging, (*VFA*) Vertebral Fracture Assessment

Biochemical parameters, normative levels: C-telopeptides (CTX) < 10–584 pg/mL; bone-specific alkaline phosphatase (bsALP) 7.6–14.9 μg/L; calcium (Ca) 8.4–10.5 mg/dL; 25-hydroxy vitamin D (25OHD); C reactive protein (CRP) < 0.5 mg/dL; erythrocyte sedimentation rate (ESR) 1–20 mm/h

### Patient 2

Patient 2 is a 52-year old male who was originally diagnosed in 2011 with a localized BRAF V600E- melanoma of the left flank, and was treated with wide local excision (Breslow thickness: 2.8 mm) and adjuvant interferon alpha. Unfortunately he developed recurrent disease in 2014 with new lung metastases, and was treated with high-dose interleukin-2 (IL-2). His disease progressed through this therapy, with the development of new osseous metastases in the axial and appendicular skeleton. He was subsequently treated with nivolumab in combination with IL-21 on a prospective clinical trial for 8 cycles of combination therapy, followed by nivolumab monotherapy. He went on to have a near complete response to ICI therapy by RECIST 1.1, with his known osseous metastases in the ribs, pelvis, femur, humerus and vertebral bodies L3 / L4 showing sclerotic change consistent with treatment response. No skeletal radiation was administered. Given his near complete response, ICI therapy was discontinued. Seven months following the cessation of therapy, the patient developed new brain metastases, pulmonary metastases, and a paraspinal metastasis at S3. The patient was treated with stereotactic radiosurgery (SRS) of the paraspinal mass and brain and was initiated on second-line dabrafenib and trametinib. After 8-months, there was an interval increase in size of the S3 paraspinal mass, and nivolumab was re-challenged. The patient went on to receive 9-months of additional ICI therapy at which time the first vertebral fracture – not associated with a metastatic lesion – was detected. The patient’s cancer was deemed to be stable is at all known sites of disease at that time. Specifically, on surveillance CT imaging, compression deformities of T2–5 were identified with new compression fractures noted at T6–12 and L1 at the time of clinic visit and vertebral fracture assessment. There was only one sclerotic lesion in the thoracic spine (T7) identified as a metastatic focus of disease; the remaining compression fractures developed in the absence of skeletal metastases. The patient’s biochemical evaluation was unremarkable. Bone density testing showed only osteopenia at the femoral neck. For treatment, he received denosumab injections every 6-months. At that time, he commenced third-line ipilimumab /nivolumab combination therapy. While the patient did not suffer additional fractures, his melanoma progressed, and he passed away 7-years after initial diagnosis.

### Patient 3

Patient 3 is a 58-year old male diagnosed with stage IV, BRAF-negative melanoma of the left ear in 2014 (stage 0) with progression to metastatic disease of the lung in 2016. The patient received first-line therapy with single agent pembrolizumab for 10-months with excellent response at which time a restaging CT indicated abnormalities of the thoracic and lumbar vertebral bodies. He carried no prior history of fracture, and no spinal metastases were identified. A comprehensive review of his outside imaging revealed an age-indeterminate T12 compression fracture sustained prior to ICI with an adjacent T11 compression deformity appearing after approximately 10 months of pembrolizumab therapy. Increased prominence of biconcave deformities of the vertebral bodies were also noted during therapy, indicating osteopenia (Fig. 1b) [17]. Given the patient’s response to therapy, pembrolizumab was discontinued after 12-months, though he was referred to the Metabolic Bone Center for continued skeletal evaluation and management. At the time of evaluation, his laboratory testing showed calcium and vitamin D deficiency. Markers of bone formation and resorption were considered normal for the patient’s sex and age and not suggestive of a high bone loss state. Bone density testing revealed only low bone density at the hip, but no frank osteoporosis. Following optimization of calcium and vitamin D status through diet and supplement, the patient retuned to clinic with updated laboratory testing. His biochemical profile indicated improved calcium and vitamin D indices as well as stable markers of bone formation and resorption. Repeat bone density testing also revealed no significant change of bone density in the hip or spine. Extensive discussion was had with the patient involving the risks and benefits of antiresorptive medications (oral / parenteral bisphosphonate vs. denosumab) in patients with vertebral fracture. He has elected to defer management beyond calcium, vitamin D and lifestyle optimization given that he is no longer taking pembrolizumab and his skeletal condition has been stable 1-year after ICI cessation. He will return to the metabolic bone center yearly for ongoing surveillance with biochemical testing and DXA imaging. In the event of ongoing bone loss or new fracture, we will revisit initiation of antiresorptive therapy. Oncologically, the patient has stable disease and has experienced neither a complete response nor progressive disease (non-CR, non-PD).

#### Resorptive bone lesions

Three patients had new destructive or resorptive appearing bony lesions that were not consistent with metastases. Two patients [4, 5] had their lesion discovered due to pain and/or swelling in the affected area prompting subsequent imaging, while patient 6 had the lesions discovered incidentally on PET scan. All three patients had concomitant inflammatory arthritis affecting separate joints with signs of systemic inflammation. Rheumatoid factor, anti-cyclic citrullinated peptide antibodies, and anti-nuclear antibodies were negative in patients 4 and 5; patient 6 did not have autoantibody testing. All patients had increased bone resorption markers (CTX, Table 2).

### Patient 4

**Fig. 2:**
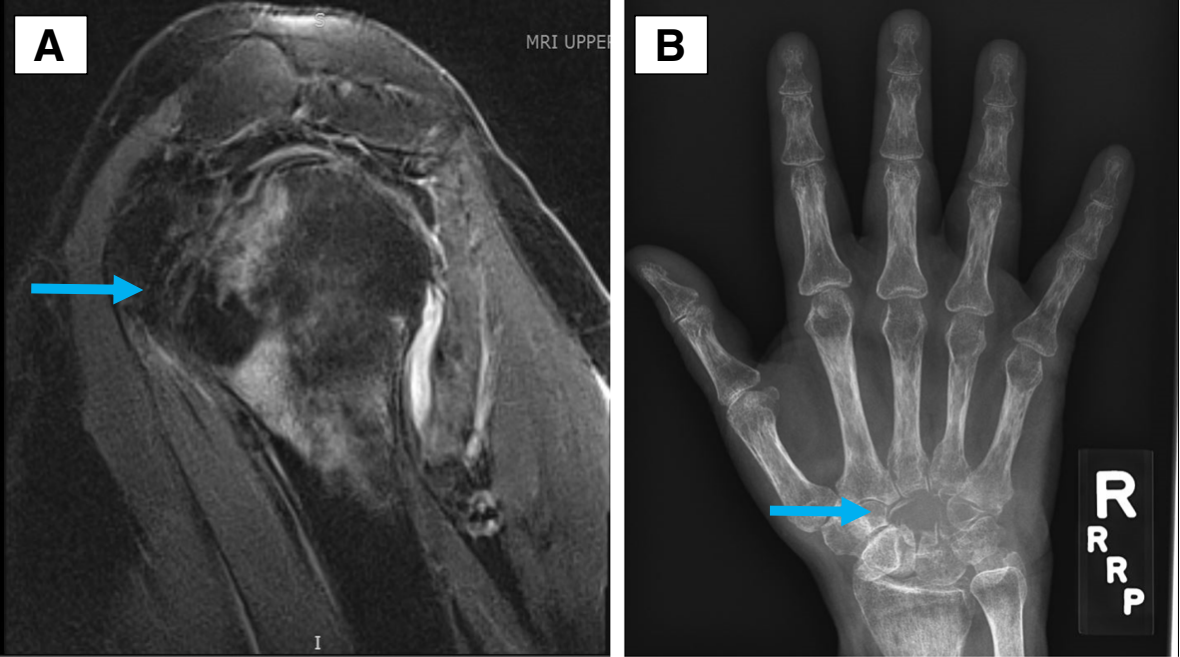
**a** Patient 4, MRI left shoulder with erosive changes of the glenohumeral articulation (arrow). **b** Patient 5, X-ray of the right hand with hamate and capitate resorption (arrow)

Patient 4 is a 59-year old male diagnosed with stage IV melanoma involving the liver only. He was treated with the first-line ipilimumab and nivolumab combination and experienced two irAEs (hypophysitis after 2-months of ICI,pneumonitis after 3-months of ICI therapy, with a second pneumonitis episode 5-months after ICI start). Eight months after ICI start, the patient developed progressive symptoms of shoulder discomfort and impaired mobility. Imaging showed a destructive lesion with surrounding bone marrow edema affecting the humeral head and the glenoid (Fig. 2a). He had extensive evaluation of his destructive shoulder lesion for potential infection or metastasis. Two separate bone biopsies showed only a mixed inflammatory infiltrate; he was started on a corticosteroid taper by his oncologist. Upon evaluation by rheumatology, his inflammatory markers were elevated; he had synovitis in the small joints of the hands and wrist, consistent with inflammatory arthritis. Based on his inflammatory arthritis, bone biopsies showing sterile inflammation and elevated inflammatory markers, he was started on therapy with adalimumab, a TNF-inhibitor. No new bony lesions developed after discontinuation of immunotherapy, and his arthritis and shoulder pain improved with adalimumab therapy. His melanoma remains in remission after 16 months of TNF-inhibitor therapy.

### Patient 5

Patient 5 is a 60-year old female who was diagnosed with stage IV clear-cell renal cell carcinoma with metastases to the lungs, brain, and bones (vertebrae, forearm). The patient was treated with first-line nivolumab and received whole brain radiation therapy, with stable disease by RECIST 1.1 after 6 doses of therapy. After 18 months of nivolumab therapy, she developed new onset right wrist swelling and stiffness. Symptoms of pain and stiffness were not severe, but a radiograph of the right wrist and hand showed resorption of two entire carpal bones and changes typical of inflammatory arthritis, namely periarticular osteopenia of metacarpophalangeal and proximal interphalangeal joints (Fig. 2b). She was briefly started on a clinical trial of nivolumab and an anti-LAG3 agent, but this was discontinued due to disease progression. Her first evaluation at our center was after starting this clinical trial. At that point, she was found to have inflammatory arthritis involving the knees, metacarpophalangeal and proximal interphalangeal joints. Per the patient’s recall, the inflammatory arthritis symptoms in joints other than the wrists started 2-months after the initial wrist swelling. Prednisone 10 mg daily was started with improvement in joint swelling, but the patient developed worsening brain metastases and entered hospice care. Further evaluation and management were not pursued in light of her progressive disease and the decision was made to transition to palliative care.

### Patient 6

Patient 6 is a 51 year old maletreated with neoadjuvant ipilimumab and nivolumab for stage II lung adenocarcinoma as part of a trial for early stage NSCLC. A pre-operative PET-CT demonstrated a new FDG avid lesion in the distal right clavicle. An MRI of this area showed bone marrow edema, joint effusion, subchondral cysts in the distal clavicle without a discrete skeletal lesion. One month later, he developed swelling and pain in his metacarpophalangeal and proximal interphalangeal joints consistent with inflammatory arthritis. He concurrently developed progressive immobility in the left elbow over a period of 6-months, leading to a fixed flexion deformity. CT imaging with IV contrast showed no metastasis, but significant joint space narrowing, sclerosis, osteophyte formation and subchondral cysts in the ulnohumeral and radio-patellar joints. Ultrasound examination of the same joint showed Doppler-positive synovitis surrounding the areas of bony hypertrophy. Unfortunately at the time of surgery he was found to have pleural metastases. The patient was subsequently treated with systemic chemotherapy for recurrent NSCLC.

## Discussion

This series describes two different skeletal phenomena observed in patients undergoing anti-PD-1 or anti-PD-1/CTLA-4 therapy for malignancy: new onset fractures and resorptive bone lesions. At first, the processes may seem discreet; however, both conditions speak to the potential influence that immune activation has on bone metabolism.

To date, there is little within the literature to explain how ICIs influence bone metabolism. Investigations into pro-inflammatory disease states confirm a direct relationship between the immune system and bone metabolism. From other studies of rheumatoid arthritis (RA), postmenopausal osteoporosis, periodontal disease and immune reconstitution with anti-retroviral therapy for the treatment of HIV, it is recognized that activated T-cells are intimately involved in skeletal remodeling [11, 18, 19]. In pro-inflammatory states such as RA that are associated with bone loss, cytokines including IFN-γ, TNF-α and IL-6 levels are increased, favoring osteoclast formation and maturation over osteoblastogenesis. In these inflammatory diseases, T-cells also produce Receptor Activator of Nuclear Factor-κB Ligand (RANK-L) and its physiological inhibitor, osteoprotegerin (OPG), the balance of which regulates osteoclastogenesis [20, 21]. Following T-cell activation, RANK-L expression is up-regulated leading to a greater ratio of RANK-L/OPG, promoting bone loss [22, 23].

Like the aforementioned conditions, ICI therapy promotes a pro-inflammatory state. In the setting of ICIs, activated T-cells secrete cytokines that assist in tumor cell destruction [24]. Cytokines implicated in promoting an anti-tumor effect include TNF-α, IL-1, − 4, − 6, IL-17 and IFN-γ. These same factors have been implicated in unfavorable skeletal remodeling states, as discussed above, where bone-resorbing osteoclasts overwhelm bone-building osteoblasts. The events described in this series represent the possible adverse effects of the pro-inflammatory environment and T-cell activation on bone metabolism due to the use of ICIs. The relevance of TNF-α, IL-6, and RANK-L in similar disease states suggests the potential utility of targeting these molecules in therapy for bone disease due to ICIs; approved drugs exist that target TNF-α, the IL-6 receptor, and RANK-L. Additionally, bisphosphonate therapy – widely used anti-osteoclastic drugs in cases of systemic osteoporosis or bone loss attributable to other disease states – could also be considered. Ultimately, additional research is needed to identify the mechanism of ICI-mediated bone loss and skeletal resorption; the pathophysiology this disease process will then drive pharmacologic intervention.

Immune checkpoint inhibitor-induced inflammatory arthritis has been described in several studies and shares some features with RA and/or spondyloarthritis (SpA) [6, 25]. Erosive disease in affected joints can occur in RA or SpA, as can generalized osteoporosis [26]. There may be parallels in the pro-inflammatory state of traditional forms of inflammatory arthritis like RA and the skeletal irAEs of immunotherapy. That inhibitors of TNF, IL1, IL6, and IL17 have all been shown to decrease the progression of bony lesions in RA and SpA further implicate these effector pathways in a proinflammatory state favoring osteoclast activation. One proposed etiology of the resorptive lesions described in this report is localized inflammation, like a sterile osteitis, leading to osteoclast activation and bone resorption.

While our study describes thought-provoking findings, it is limited by its retrospective nature and limited sample size. Included patients were only those referred to physicians in endocrinology or rheumatology for skeletal events identified due to symptomatic presentation or opportunistic imaging. Patients with asymptomatic bone loss, occult and progressive fractures, or mild lesions may not have prompted endocrinology or rheumatology referral. The included patients had different demographic profiles, tumor types, varying stages of disease and were receiving different therapeutic ICI regimens at the time of their skeletal event, presenting challenges in drawing clear associations between these factors the development of skeletal irAEs. Because laboratory and imaging studies were obtained for the purposes of clinical care rather than a research protocol, not all patients had the same evaluation. For example, in cases of fracture detected in CT surveillance imaging for routine oncologic management, DXA evaluation followed for a general assessment of BMD; vertebral fracture assessment was obtained, where appropriate, to best characterize compromised vertebral bodies. In cases of focal skeletal resorption with localized bone and joint symptoms, MRI was the imaging modality of choice for diagnostic assessment. We anticipate that as additional cases of skeletal AEs arise in the setting of ICI, such findings will drive specific imaging and biochemical work-up protocols tailored to the presenting symptoms and degree of suspicion for fracture or resorptive lesion.

## Conclusion

Despite the limited numbers, our observations support the identification of a new class of skeletal-related irAEs. Future areas of study for these newly appreciated clinical phenomena will also include assessment of risk factors for development of skeletal irAEs. Such risk factors might include pre-existing osteoporosis/osteopenia, fragility fractures, concomitant inflammatory arthritis or other autoimmune sequelae. Genetic or environmental factors and their role in the skeletal sequelae of immunotherapy may also bear consideration. In addition, specification of generalized bone loss leading to fracture vs. focal bone resorption will need to be undertaken. As our clinical experience with ICIs expands and irAE prevalence increases, additional bony manifestations may be identified, though additional research is required.

## Abbreviations

CTLA-4: Cytotoxic T-lymphocyte associated protein 4
CTX: C-telopeptides
DXA: Dual energy X-ray absorptiometry
ICI: Immune checkpoint inhibitor
IL-2: Interleukin-2
irAEs: Immune-related adverse events
OPG: Osteoprotegerin
PD-1: Programmed death-1
RANK-L: Receptor Activator of Nuclear Factor-κB Ligand
SpA: Spondyloarthritis
